# Application patterns and outcomes of hematopoietic stem cell transplantation in peripheral T-cell lymphoma patients: a multicenter real-world study in China

**DOI:** 10.1186/s40164-024-00557-9

**Published:** 2024-08-24

**Authors:** Hongye Gao, Zhuoxin Zhang, Jiali Wang, Yannan Jia, Yawei Zheng, Xiaolei Pei, Weihua Zhai, Rongli Zhang, Xin Chen, Qiaoling Ma, Jialin Wei, Donglin Yang, Aiming Pang, Yi He, Sizhou Feng, Hao Zhang, Xin Du, Xianmin Song, Yao Liu, Dehui Zou, Erlie Jiang

**Affiliations:** 1 State Key Laboratory of Experimental Hematology, National Clinical Research Center for Blood Diseases, Haihe Laboratory of Cell Ecosystem, Institute of Hematology & Blood Diseases Hospital, Chinese Academy of Medical Sciences & Peking Union Medical College, Tianjin, China; 2 Tianjin Institutes of Health Science, Tianjin, China; 3 Department of Hematology, Shanghai General Hospital, Shanghai Jiao Tong University School of Medicine, Shanghai, China; 4 Department of Hematology, Affiliated Hospital of Jining Medical University, Jining, China; 5 Department of Hematology and Shenzhen Bone Marrow Transplantation Public Service Platform, Shenzhen Second People’s Hospital, The First Affiliated Hospital of Shenzhen University, Shenzhen, China; 6 Department of Hematology Oncology, Chongqing Key Laboratory of Translational Research for Cancer Metastasis and Individualized Treatment, Chongqing University Cancer Hospital, Chongqing, China

**Keywords:** Hematopoietic stem cell transplantation, Peripheral T-cell lymphoma, Autologous HSCT, Allogeneic HSCT

## Abstract

The optimal timing and type of hematopoietic stem cell transplantation (HSCT) for treating peripheral T-cell lymphoma (PTCL) remain controversial. This retrospective real-world study investigated the application pattern and outcomes of HSCT in China. The analysis encompassed 408 PTCL patients with a median age of 45.5 years, all of whom received initial adequate therapy at five hospitals. Among patients with nodal PTCL who responded effectively to first-line therapy (the “responders”, *n* = 127) and subsequently underwent HSCT consolidation (*n* = 47, 37.0%), 93.6% received auto-HSCT, while 6.4% underwent allo-HSCT. Front-line auto-HSCT showed potential for long-term disease control in nodal PTCL responders. Among non-nodal PTCL responders (*n* = 80) with HSCT (*n* = 26, 32.5%), 46.2% underwent allo-HSCT and 53.8% received auto-HSCT. Upfront allo-HSCT provides longer progression-free survival (PFS) for non-nodal PTCL responders, with lower 3-year cumulative incidence of relapse (CIR) (16.7% vs. 56.0%) and comparable non-relapse mortality (NRM) (10.4% vs. 11.0%) compared to auto-HSCT. For patients who achieved remission with second-line salvage regimens, allo-HSCT was the primary choice (82.4%) for non-nodal PTCL, while auto-HSCT was more common (82.4%) in nodal PTCL. Nodal PTCL patients underwent auto-HSCT after ≥ 3 lines of treatment had a higher 3-year CIR (81.0%) compared to those treated in the first (26.0%) or second line (26.0%). Non-nodal PTCL patients underwent allo-HSCT after ≥ 3 lines had a higher 3-year NRM (37.5%) compared to after first (10.4%) or second line treatment (8.5%). These findings highlight distinct HSCT application patterns for PTCL in China, emphasizing the impact of early disease control and upfront consolidative HSCT.

**Supplementary Information:**

The online version contains supplementary material available at 10.1186/s40164-024-00557-9.

**To the editor,**

Peripheral T-cell lymphoma (PTCL) presents significant treatment challenges due to its heterogeneous nature and generally poor prognosis [1]. Hematopoietic stem cell transplantation (HSCT) offers a potential cure for PTCL. However, the optimal timing and type of transplant, whether autologous (auto-HSCT) or allogeneic (allo-HSCT), are still under debate.

This retrospective, real-world study has been conducted at five HSCT-qualified medical centers in China to investigate the impact of HSCT. After rigorously screening, we further analyzed 408 PTCL patients who had received adequate initial treatment and had confirmed response status (median age: 45.5 years).

Consolidative auto-HSCT after first-line treatment of PTCL has been extensively published [2–6]. However, due to the diverse subtypes of PTCL and the varying patient characteristics across different studies, the conclusions remain controversial. In the present study, auto-HSCT was the preferred HSCT type for 93.6% of nodal PTCL responders (Additional file 1, Table S1), including those with complete remission (CR) or satisfactory partial remission (PR) (Fig. 1A-C; Fig. S1). The progression-free survival (PFS) and overall survival (OS) curves of patients with auto-HSCT reached plateau (Fig. 1D; Fig. S2A), suggesting auto-HSCT may achieve long-lasting response and even cure [2, 5, 6]. The benefit of auto-HSCT consolidation on PFS for nodal-PTCL responders was also observed when excluding ALK + ALCL, also in the PSM cohort (Fig. S2B, Fig. S3A and Table S4).

**Fig. 1:**
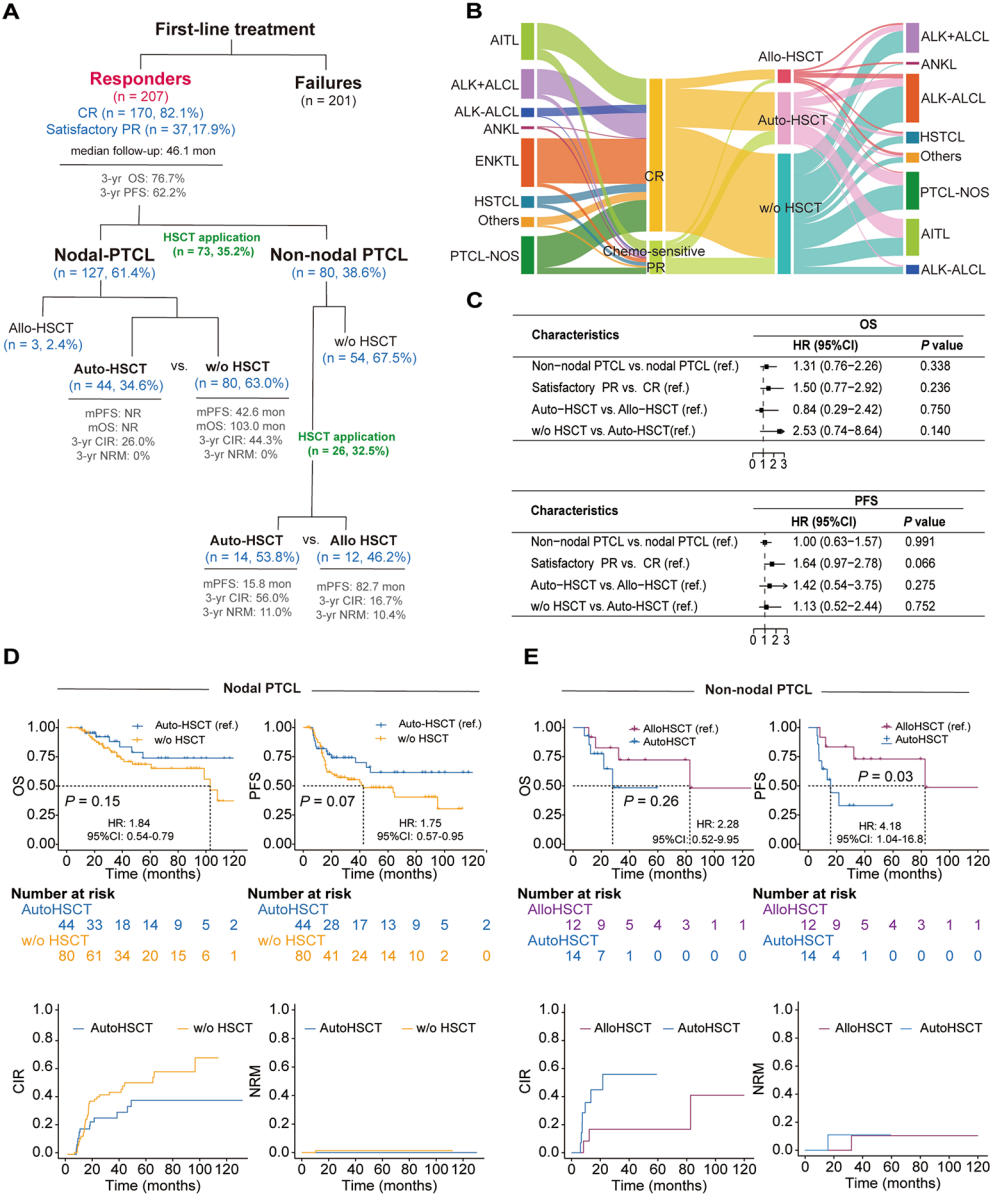
Flow chart and treatment patterns for patients who responded effectively to first-line treatment. (**A**) Flow chart for patients with PTCL who demonstrated a positive response (responders) to first-line treatment. (**B**) Initial treatment response and subsequent treatment choices in responders. (**C**) Analysis of clinical characteristics impacting progression-free survival (PFS) and overall survival (OS) in responders using a univariate Cox model. (**D**) Outcomes since initial treatment with autologous hematopoietic stem cell transplantation (auto-HSCT) and without HSCT in nodal-PTCL responders. (**E**) Outcomes for non-nodal PTCL responders with auto-HSCT and allogeneic-HSCT (allo-HSCT). For PR patients, if the initial treatment was deemed insufficient by the hematologist and immediate salvage therapy was needed, it was considered unsatisfactory PR. Otherwise, it was classified as satisfactory PR to distinguish between responsive patients and those with primary refractory disease. CIR: Cumulative incidence of relapse; NRM: Non-relapse mortality; w/o: Without; CI: Confidence Interval; AITL: Angioimmunoblastic T-cell lymphoma; ALK-ALCL: Anaplastic lymphoma kinase-negative anaplastic large cell lymphoma; ALK + ALCL: Anaplastic lymphoma kinase-positive anaplastic large cell lymphoma; ANKL: Aggressive NK-cell leukemia; ENKTL: Extranodal NK/T-cell lymphoma, nasal type; HSTCL: Hepatosplenic T-cell lymphoma; PTCL-NOS: Peripheral T-cell lymphoma, not otherwise specified

Previous studies have shown that up-front allo-HSCT in PTCL is associated with a low relapse rate but a high risk of non-relapse mortality (NRM) [7, 8]. In our analysis for non-nodal PTCL who underwent HSCT consolidation (*n* = 26; Fig. S1D; Fig. S2C and D), 46.2% of patients underwent allo-HSCT, while 53.8% auto-HSCT (Fig. 1A). Among these patients, those who underwent allo-HSCT demonstrated a more favorable PFS (median PFS: 82.7 months vs. 15.8 months, *P* = 0.031; Fig. 1E). Additionally, the 3-year CIR and NRM were 16.7% and 10.4% for the allo-HSCT group, and 56.0% and 11.0% for the auto-HSCT group. The lower NRM was also confirmed in the PSM cohort of non-nodal responders (Fig. S3B and Table S5). These results suggest that upfront allo-HSCT may be associated with a lower CIR while maintaining comparable NRM rates compared to auto-HSCT in non-nodal PTCL patients.

The optimal HSCT consolidation strategy for patients in remission following salvage therapy remains uncertain in the literature [9, 10]. While both auto-HSCT and allo-HSCT are considered viable options, there is a lack of comparative data and varying transplant preferences among centers, influenced by factors such as transplant eligibility, pathological subtypes and disease risk stratification. This study observed a distinct HSCT pattern after second-line treatment, with non-nodal PTCL patients more likely to undergo allo-HSCT (82.4%) and nodal PTCL patients predominantly choosing auto-HSCT (82.4%; Fig. 2A and Table S2). This finding highlights it is challenging to compare the efficacy of auto-HSCT and allo-HSCT after salvage therapy for PTCL, due to the selection propensity in the type of HSCT for different PTCL subtypes.

**Fig. 2:**
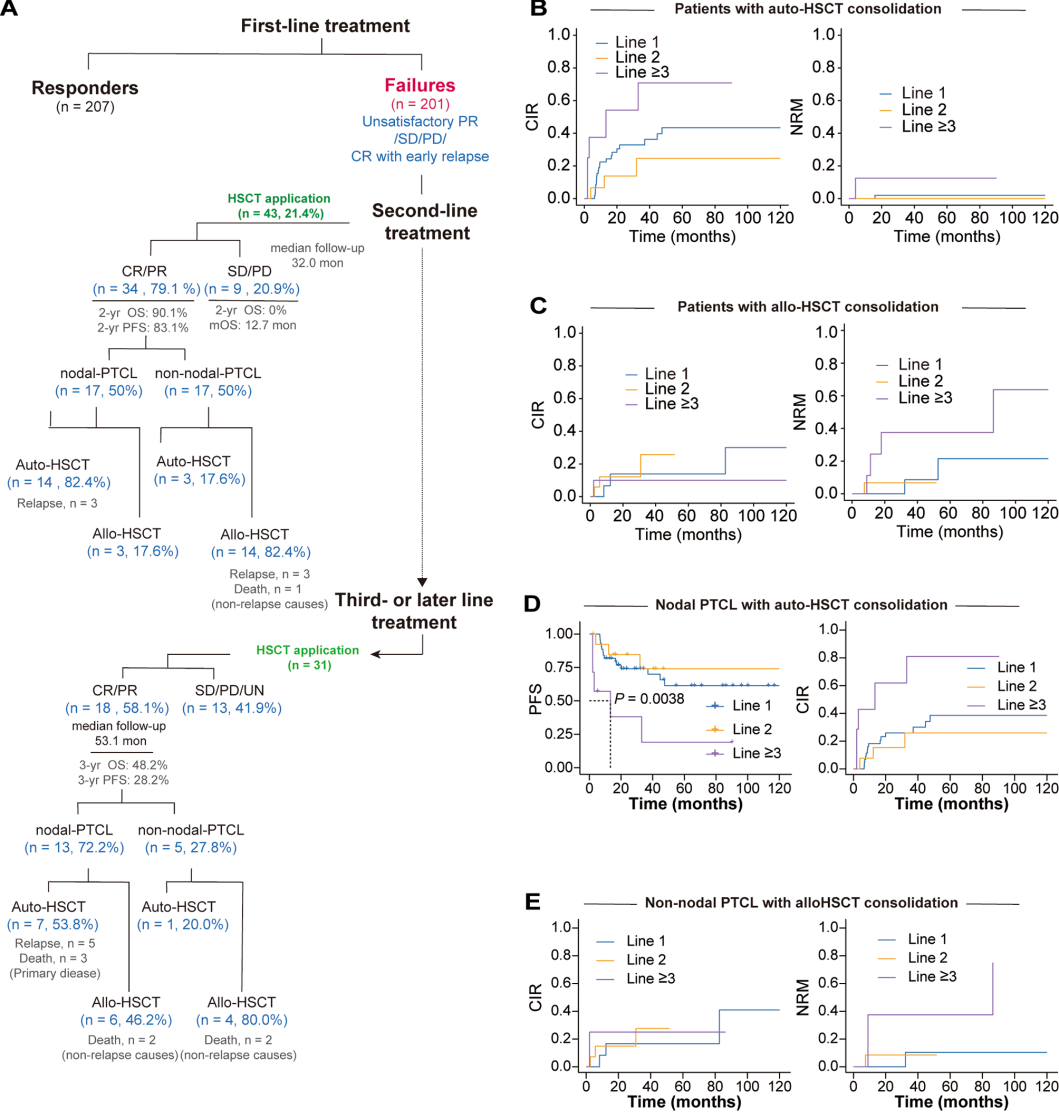
Flow chart for non-responders and HSCT application outcomes at the different lines. (**A**) Flow chart for patients with PTCL who did not respond effectively to first-line treatment. (**B**) CIR and NRM following auto-HSCT for patients who achieved remission at first-line, second-line, and third-line treatment. (**C**) CIR and NRM following allo-HSCT consolidation for patients in remission at first-line, second-line, and third-line treatment. (**D**) PFS and CIR following auto-HSCT for nodal-PTCL patients with remission status at first-line, second-line, and third- or later-line treatment. (**E**) CIR and NRM following allo-HSCT for non-nodal PTCL patients with remission status at first-line, second-line, and third- or later-line treatment

Our findings also indicate that HSCT performed after ≥ 3 lines treatment was associated with adverse outcomes (Fig. 2B-E, Fig. S8; Table S3). Specifically, nodal PTCL patients in remission status who underwent auto-HSCT after ≥ 3 lines showed a significantly higher 3-year CIR at 81.0%, compared to 26.0% in the first line and 26.0% in the second line (Fig. 2D). One possible reason for the reduced effectiveness of later-line auto-HSCT is the resistance to high-dose chemotherapy in patients who failed front-line treatment [11].

For non-nodal patients, the application of allo-HSCT consolidation following ≥ 3 lines treatment demonstrated a significant increase in 3-year NRM rates, reaching 37.5% in comparison to 10.4% in the first and 8.5% in the second-line treatment, although with a comparable 3-year CIR (Fig. 2E). This finding emphasizes the impact of a heavy treatment history on bone marrow hematopoiesis and immune reconstitution in patients undergoing allo-HSCT, rendering them more vulnerable to complications such as graft-versus-host disease and infections [8, 12].

Overall, our study underscores the distinct HSCT applications for nodal and non-nodal PTCL in China, highlighting the potential drawbacks of consolidative HSCT in later-line treatment. Further research with larger sample sizes is warranted to confirm our findings.

### Electronic supplementary material

Below is the link to the electronic supplementary material.

Supplementary Material 1

## Data Availability

The datasets and analysis codes are available on reasonable request to the corresponding author.

## Abbreviations

HSCT: Hematopoietic Stem Cell Transplantation
Auto-HSCT: Autologous Hematopoietic Stem Cell Transplantation
Allo-HSCT: Allogeneic Hematopoietic Stem Cell Transplantation
ALK + ALCL: Anaplastic Lymphoma Kinase-Positive Anaplastic Large Cell Lymphoma
CIR: Cumulative Incidence of Relapse
NRM: Non-Relapse Mortality
CR: Complete Remission
PR: Partial Remission
SD: Stable Disease
PD: Progressive Disease
