# Supplementary material for: Application patterns and outcomes of hematopoietic stem cell transplantation in peripheral T-cell lymphoma patients: a multicenter real-world study in China
Source: Exp Hematol Oncol. 2024 Aug 24;13:88. doi: 10.1186/s40164-024-00557-9 (PMC11344441; doi:10.1186/s40164-024-00557-9)
Supplement: Supplementary file 1 — Supplementary Material 1 [file 40164_2024_557_MOESM1_ESM.docx]

**Application Patterns and Outcomes of Hematopoietic Stem Cell Transplantation in Peripheral T-Cell Lymphoma Patients: A Multicenter Real-world Study in China**

Gao et. al

**Additional File 1 Content**

[Patients and Methods 2](#_Toc172396838)

[Figure S1. Survival outcomes and treatment comparisons in patients who effectively respond to the initial treatment 5](#_Toc172396839)

[Figure S2. Survival outcomes and treatment comparisons in nodal patients and non-nodal patients 6](#_Toc172396840)

[Figure S3. Survival outcomes for the matched cohort of PTCL with nodal and non-nodal subtypes 8](#_Toc172396841)

[Figure S4. Survival outcomes for HSTCL and ENKTL 9](#_Toc172396842)

[Figure S5. Impact of bone marrow involvement factor in patients with auto-HSCT 10](#_Toc172396843)

[Figure S6. Disease response and subsequent HSCT application patterns 11](#_Toc172396844)

[Figure S7. Outcomes in HSCT patients with remission and non-remission status post-second-line treatment 12](#_Toc172396845)

[Figure S8. Outcomes following HSCT for patients at different treatment lines 13](#_Toc172396846)

[Table S1. Patients' baseline characteristics for the first-line treatment effectively responders 15](#_Toc172396847)

[Table S2. Patients' baseline characteristics for the first-line treatment non-responders 18](#_Toc172396848)

[Table S3. Patients' baseline characteristics for HSCT after the third-line and beyond treatment 20](#_Toc172396849)

[Table S4. Baseline for PTCL responders with nodal subtypes before and after propensity score matching 22](#_Toc172396850)

[Table S6. Non-relapse mortality (NRM) rates of allo-HSCT in treating PTCL according to the literature review 26](#_Toc172396851)

# Patients and Methods

**Study design and patients**

This retrospective multicenter study was approved from the Ethics Committee of the Institute of Hematology & Blood Diseases (Approval Number: QTJC2024018-EC-1) and a waiver of informed consent.

Patients aged 18 years or older diagnosed with PTCL according to World Health Organization criteria [1] **between January 2005 and June 2023** were identified from **5 qualified clinical centers** in China. The data of 497 patients were collected, with some patients being part of the National Longitudinal Cohort of Hematological Diseases (NICHE) cohort (NCT04645199). The data comprised baseline patient characteristics, histological subtypes, International Prognostic Index (IPI) [2], and Prognostic Index for T-cell lymphoma (PIT) scores [3], treatment details, response to treatment, and follow-up information.

In this study, we categorized PTCL into nodal and non-nodal group. The treatment response was evaluated according to the Lugano 2014 criteria [4]. Bone marrow involvement was assessed by bone marrow biopsy and aspiration. Flow cytometry was also performed on bone marrow aspirate samples to confirm the presence of lymphoma cells and assess for aberrant phenotypes.

We excluded patients who met any of the following criteria: death during first-line treatment due to causes unrelated to their primary disease (e.g., cardiovascular events), inability to complete the first-line induction therapy, an indeterminate initial treatment response, or loss to follow-up following initial treatment.

**Treatment response to the initial therapy**

We referred to the term “unsatisfactory PR” as described by Bellei et al’s work [5]. In our study, two experienced hematologists were invited to re-evaluate the response to frontline treatment, particularly the status of PR, based on clinical reports. For PR patients, if either hematologist determined the first-line treatment was inadequate, requiring immediate salvage therapy, we classified the case as unsatisfactory PR. Otherwise, it was categorized as satisfactory PR. This definition was introduced to better differentiate patients responsive to chemotherapy from those with primary refractory disease.

Patients were further categorized based on their response to initial therapy: First-line treatment effective responders (hereinafter referred to as responders), consisting of those who achieved complete remission (CR) or satisfactory PR after their initial treatment. Patients who did not respond adequately to initial treatment, including those with refractory disease (SD/PD/unsatisfactory PR) or early relapse (within 2 months after completing first-line treatment), were categorized as first-line treatment failures (non-responders).

**Endpoint definitions**

The study's primary outcomes included progression-free survival (PFS), overall survival (OS), non-relapse mortality (NRM), and cumulative incidence of relapse (CIR). PFS was defined as the time to the first occurrence of disease progression, relapse, or death. OS indicated the time to death from any cause. NRM was characterized as death from any cause without evidence of lymphoma relapse or progression. For the responders, PFS, OS, NRM, and CIR were calculated from the commencement of initial treatment. For those who did not respond adequately to initial treatment, PFS, OS, CIR, and NRM were calculated from the initiation of HSCT.

**Statistical analysis**

OS and PFS were estimated using the Kaplan-Meier method, and group comparisons were conducted using the log-rank test. CIR and NRM were assessed by the cumulative incidence function, accounting for competing risks, and compared by Fine-Gray's test [6]. Nelson-Aalen cumulative risk survival curves were generated to visually depict the outcomes related to relapse and NRM.

The research employed the Cox proportional hazards model to determine the hazard ratio (HR) and corresponding 95% confidence intervals (CI) for both OS and PFS. Propensity score matching (PSM) was performed to minimize potential confounding factors and achieve balanced covariates between groups. Covariates were selected based on previous literature and clinical experience.

One-to-one nearest neighbor matching without replacement was conducted using the “Matching package” in R [7].

All statistical analyses were two-tailed, with statistical significance assessed at the *P* < 0.05 level. Data analysis was performed by R version 4.3.2.(<https://cran.r-project.org/>).

# Figure S1. Survival outcomes and treatment comparisons in patients who effectively respond to the initial treatment


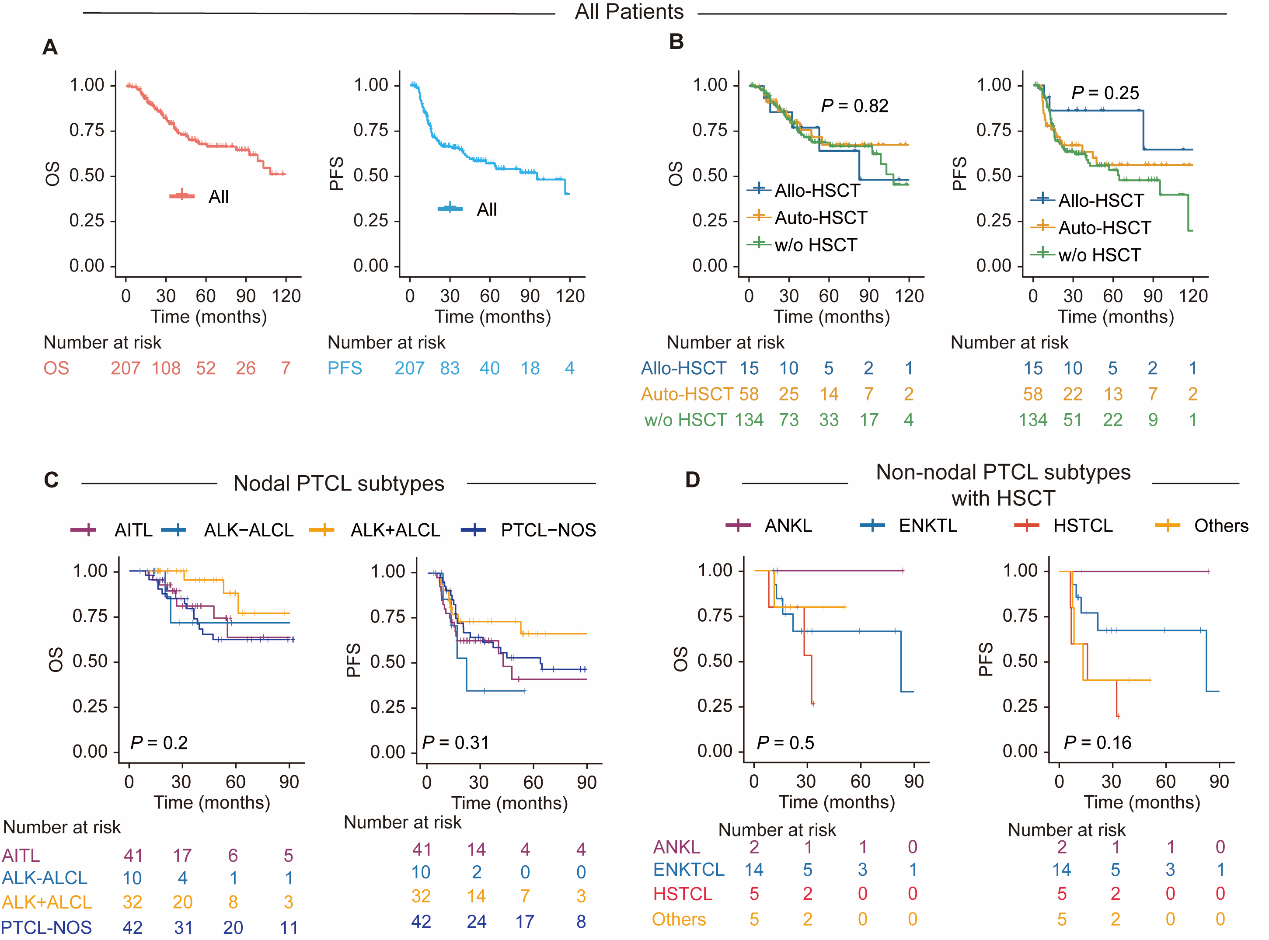


Overall survival (OS) and progression-free survival (PFS) data for the following patient cohorts: All patients who effectively responded to initial treatment (responders) since diagnosis (A); The cohort stratified by the treatments received following first-line therapy, including those who underwent HSCT and those who did not (B). The responders with nodal PTCL subtypes (C) and with non-nodal PTCL subtypes who underwent HSCT (D).

# Figure S2. Survival outcomes and treatment comparisons in nodal patients and non-nodal patients


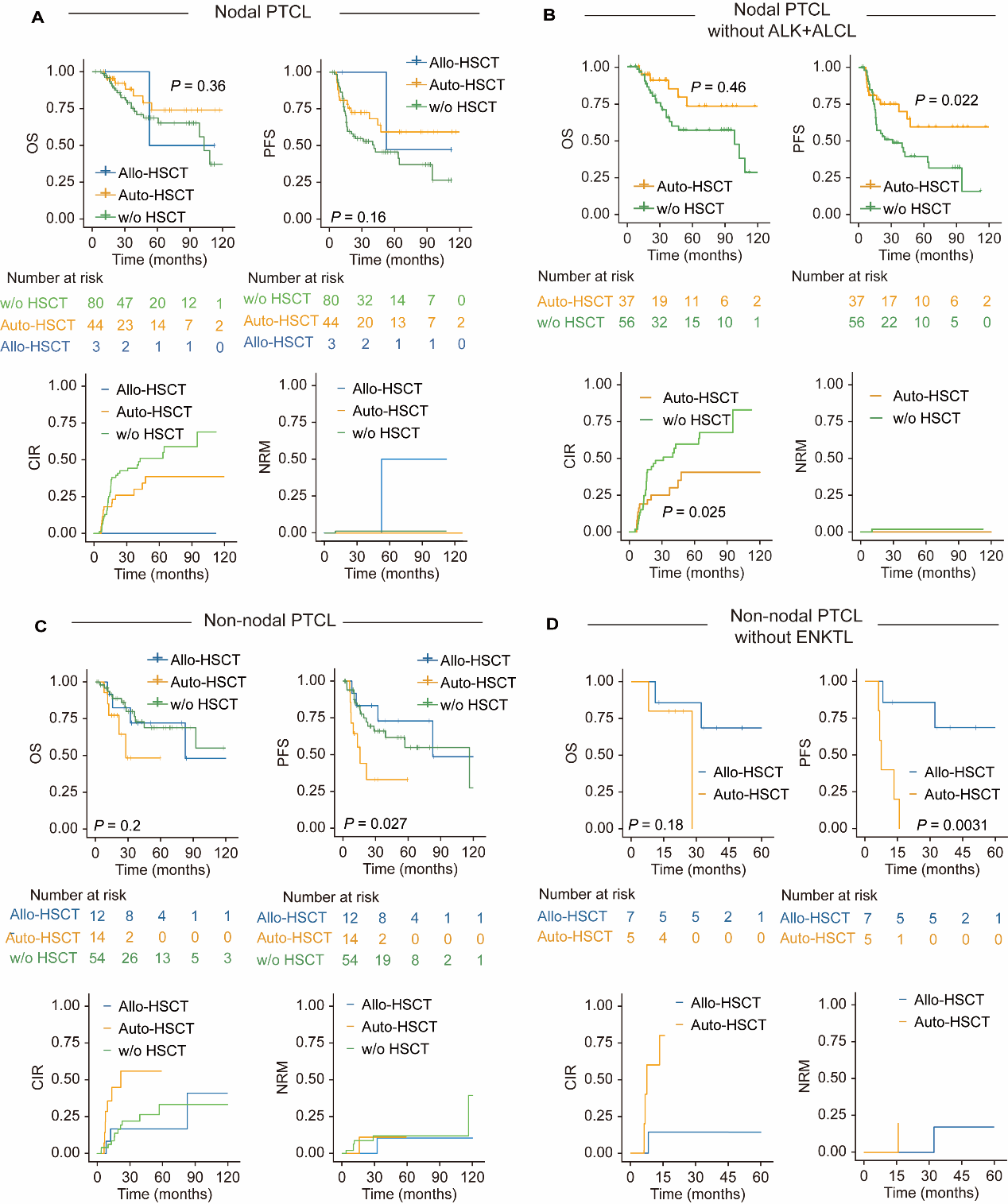


Survival outcomes, including overall survival (OS), progression-free survival (PFS), cumulative incidence of relapse (CIR), and non-relapse mortality (NRM), were analyzed for responders with nodal (A) and non-nodal PTCL (C). These outcomes were further stratified by auto-HSCT, allo-HSCT and non-HSCT groups. Survival outcomes were compared for nodal PTCL excluding the ALK+ anaplastic large cell lymphoma (ALCL) subtype (B) and for non-nodal PTCL excluding the extranodal NK/T-cell lymphoma (ENKTL) subtype (D).

# Figure S3. Survival outcomes for the matched cohort of PTCL with nodal and non-nodal subtypes


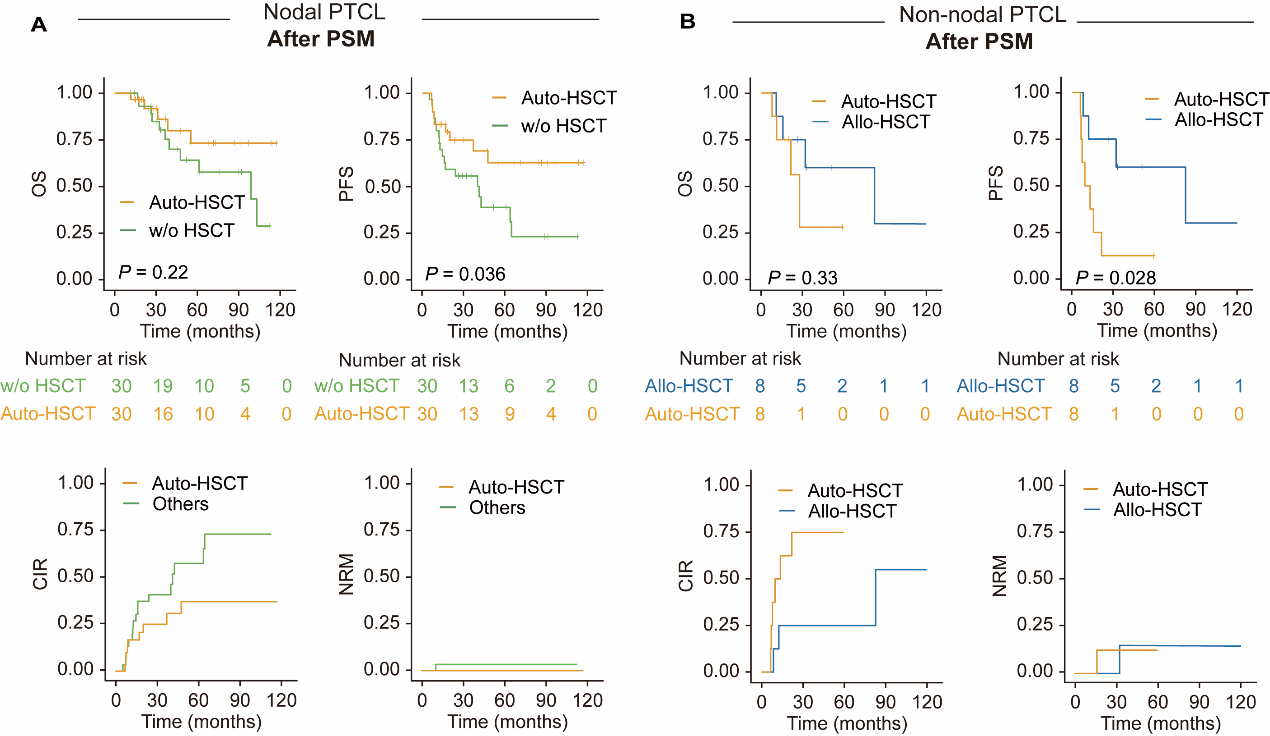


Considering the potential baseline differences among the different treatment groups in responders with nodal PTCL and non-nodal PTCL (Fig. 1D and E), we conducted propensity score matching (PSM) methods to balance the clinical characteristics between the comparison groups. The outcomes, including OS, PFS, CIR, and NRM, were analyzed in responders with nodal PTCL and non-nodal PTCL after first-line treatment post-PSM (A and B). For detailed information on the PSM methods, please refer to Tables S4 and S5.

# Figure S4. Survival outcomes for HSTCL and ENKTL


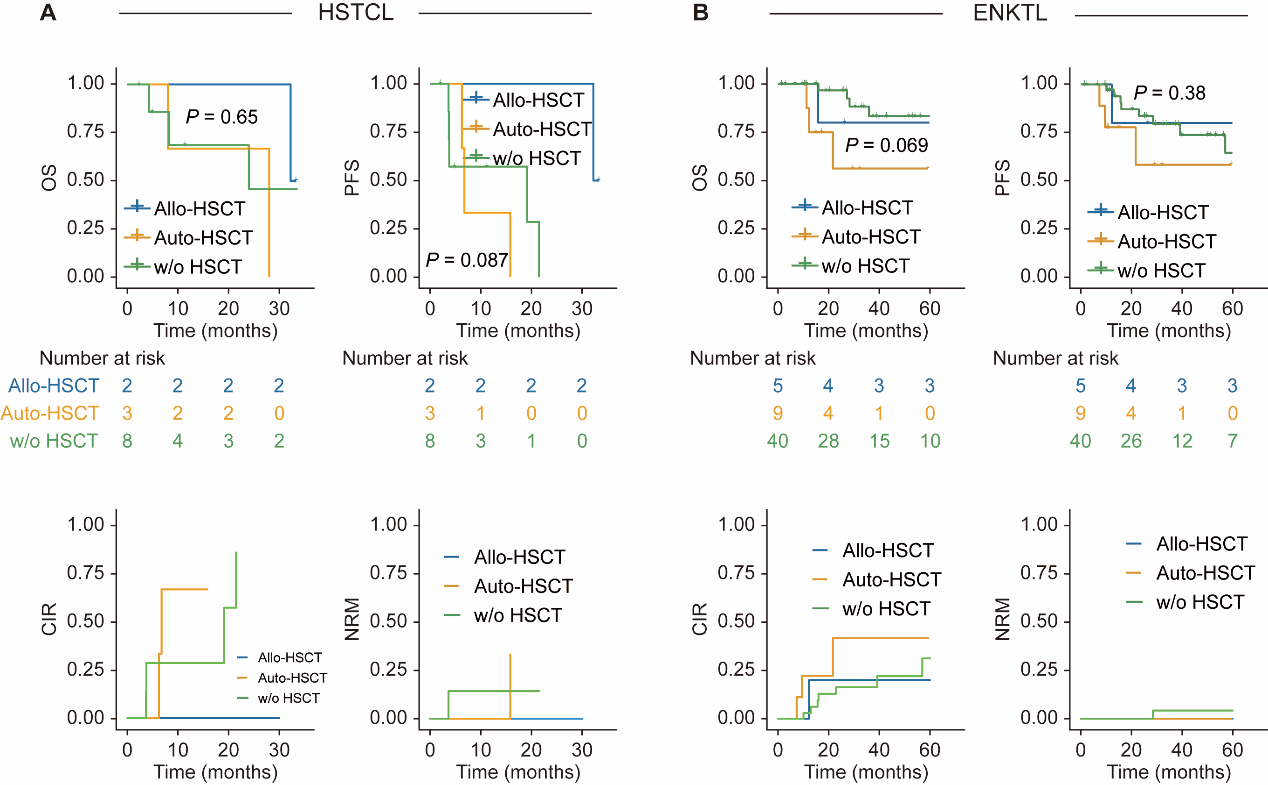


Comparison of outcomes (OS, PFS, CIR and NRM) between different treatments after the first-line treatment among responders with Hepatosplenic T-cell lymphoma (HSTCL; A) and ENKTL; B).

# Figure S5. Impact of bone marrow involvement factor in patients with auto-HSCT


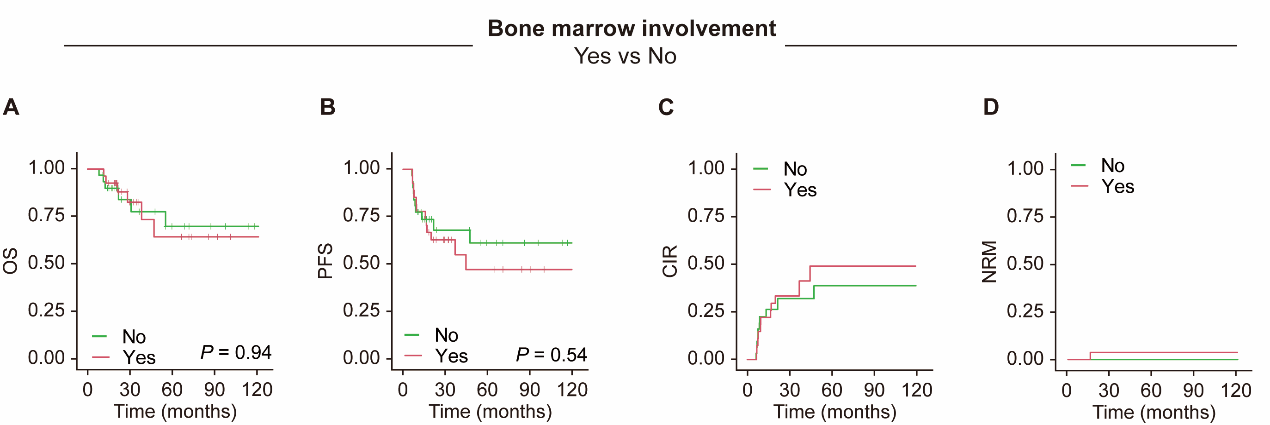


OS (A), PFS (B), CIR (C) and NRM (D) for patients receiving upfront consolidative auto-HSCT who presented with (n = 27) and without (n = 31) bone marrow involvement at baseline

# Figure S6. Disease response and subsequent HSCT application patterns


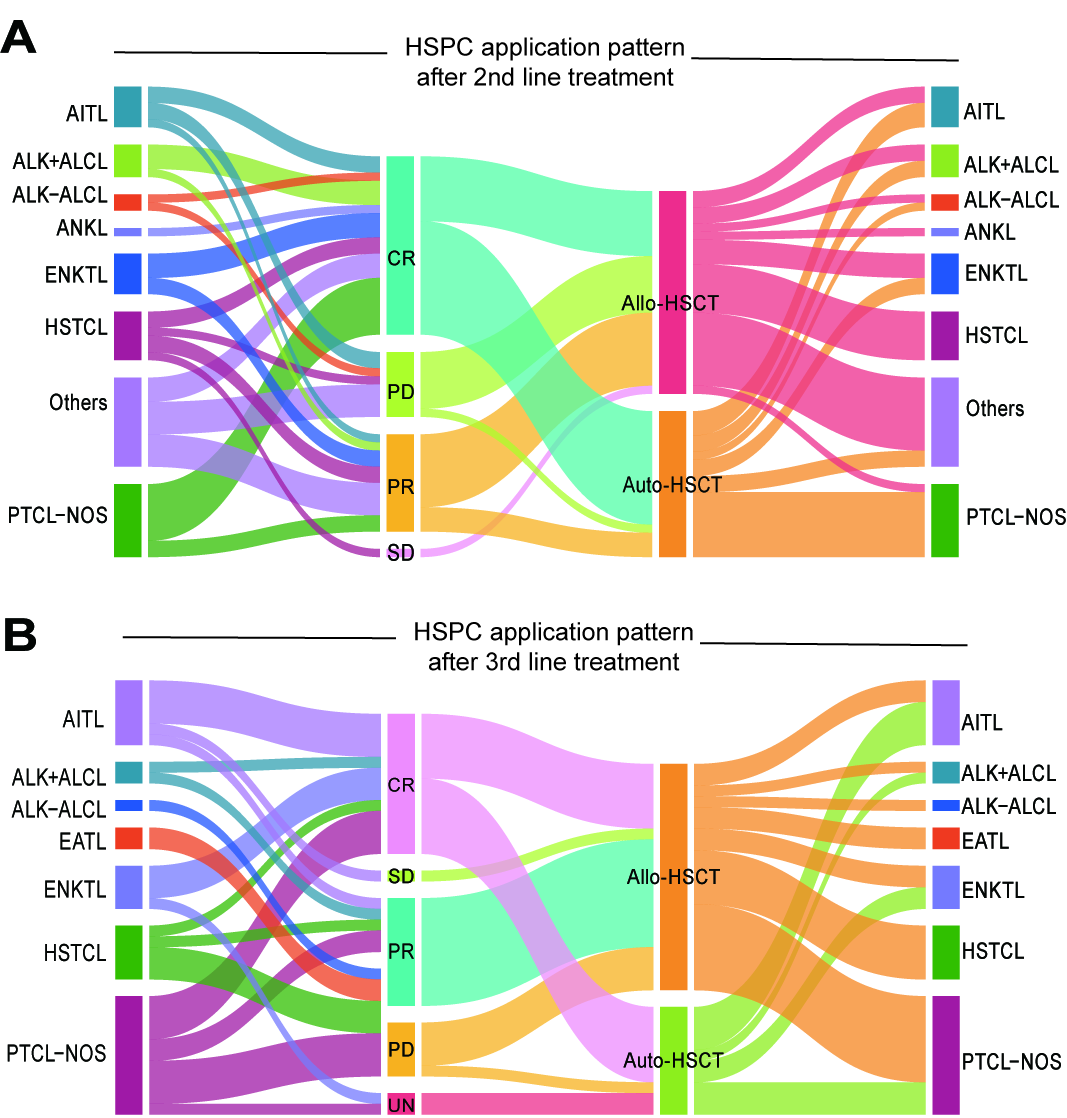


Disease status before HSCT and the modalities of HSCT in patients who have undergone either a second-line treatment (A) or more than three lines of treatment (B).

# Figure S7. Outcomes in HSCT patients with remission and non-remission status post-second-line treatment


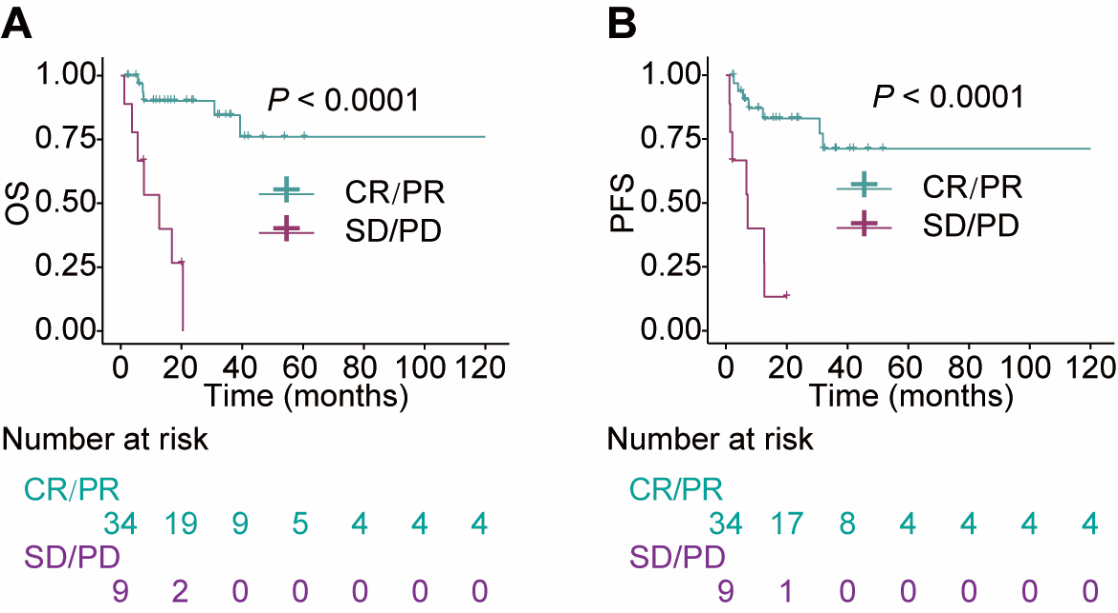


Comparison of OS (A) and PFS (B) in patients who underwent HSCT after second-line treatment, comparing those who achieved remission before HSCT with those who did not.

# Figure S8. Outcomes following HSCT for patients at different treatment lines


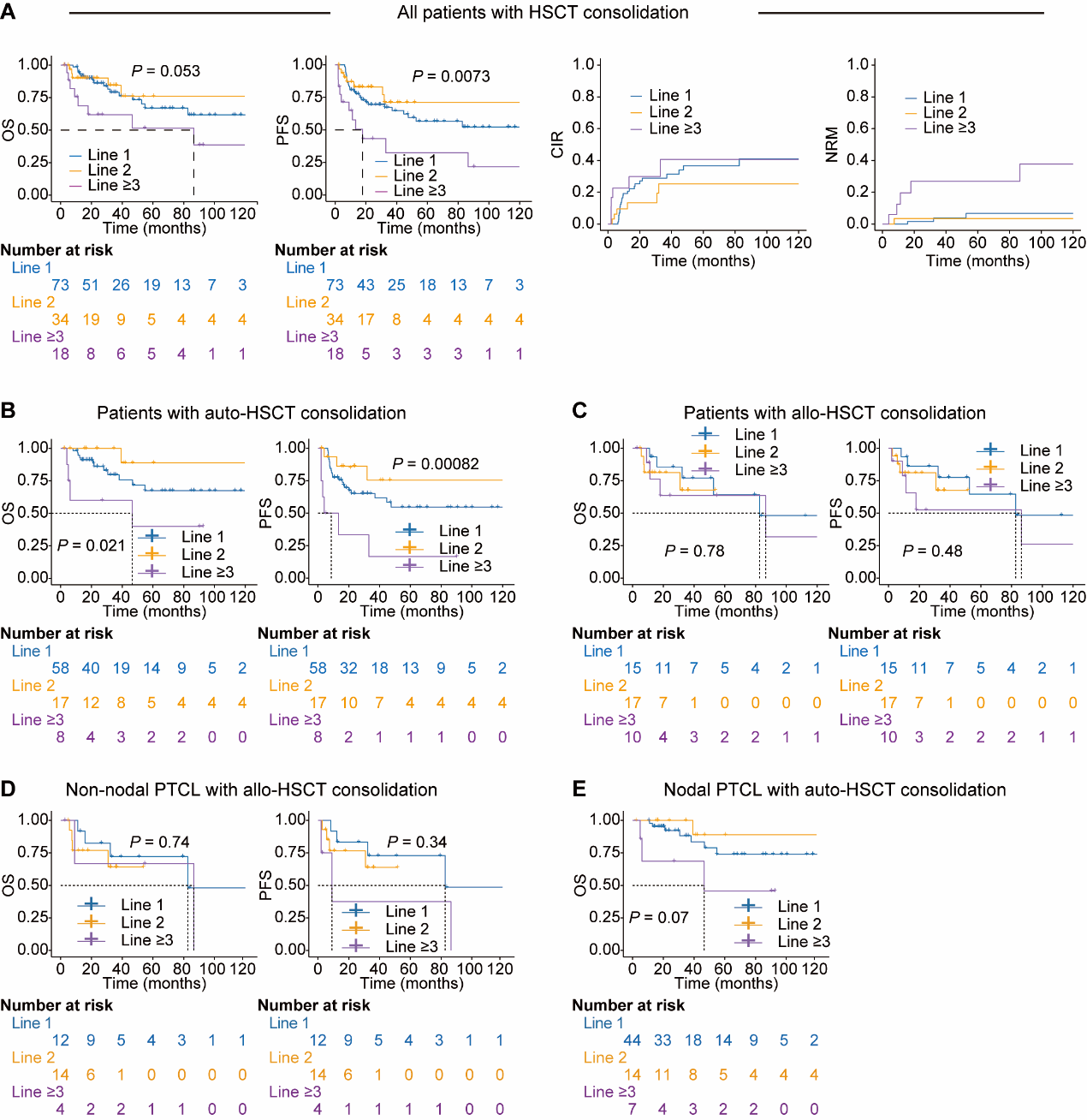


(A) Outcomes (OS, PFS, CIR and NRM) following HSCT consolidation (both auto-HSCT and allo-HSCT) for patients achieved remission at first-line, second-line, and third or subsequent lines treatment. (B) OS and PFS following auto-HSCT for patients with remission status at first line, second line, and third or subsequent lines treatment. (C) OS and PFS following allo-HSCT for patients with remission status at first-line, second-line, and third or subsequent lines treatment. (D) PFS and OS following allo-HSCT for non-nodal patients with remission status at first-line, second-line, and third or subsequent lines treatment. (E) OS following auto-HSCT for nodal-PTCL patients with remission status at first-line, second-line, and third or subsequent lines treatment.

# Table S1. Patients' baseline characteristics for the first-line treatment effectively responders

| **Characteristics** | **Overall**  n = 207 | **Nodal-PTCL**  n = 127 | **Non-nodal PTCL**  n = 80 | ***P* value** |
| --- | --- | --- | --- | --- |
| **Male (%)** | 129 (62.3) | 83 (65.4) | 46 (57.5) | 0.323 |
| **Age at diagnose, median (IQR), years** | 45 (32-58) | 46 (34-60) | 45 (29-54) | 0.073 |
| **Disease (%)** |  |  |  |  |
| AITL | 41 (19.8) | 41 (32.3) | — |  |
| ALK-ALCL | 10 (4.8) | 10 (7.9) | — |  |
| ALK+ALCL | 33 (15.9) | 33 (26.0) | — |  |
| PTCL-NOS | 42 (20.3) | 42 (33.1) | — |  |
| ANKL | 3 (1.4) | — | 3 (3.8) |  |
| ENKTL | 54 (26.1) | — | 54 (67.5) |  |
| HSTCL | 13 (6.3) | — | 13 (16.2) |  |
| Others* | 11 (5.3) | 1 (0.8) | 10 (12.5) |  |
| **IPI score > 2 (%)** | 62 (30.0) | 40 (31.5) | 22 (27.5) | 0.649 |
| **PIT risk factors (%)** |  |  |  | 0.188 |
| 0-1 | 137 (66.2) | 78 (61.4) | 59 (73.8) |  |
| 2 | 47 (22.7) | 33 (26.0) | 14 (17.5) |  |
| 3-4 | 23 (11.1) | 16 (12.6) | 7 (8.8) |  |
| **Involvement of the spleen (%)** |  |  |  | 0.703 |
| No | 154 (74.4) | 95 (74.8) | 59 (73.8) |  |
| Yes | 52 (25.1) | 31 (24.4) | 21 (26.2) |  |
| UN | 1 (0.5) | 1 (0.8) | 0 (0.0) |  |
| **Involvement of the bone marrow (%)** | 80 (38.6) | 51 (40.2) | 29 (36.2) | 0.678 |
| **Involvement of the CNS (%)** | 2 (1.0) | 1 (0.8) | 1 (1.2) | > 0.999 |
| **Frontline treatment regimens (%)** |  |  |  | **0.001** |
| CHOP-like without novel agents | 10 (23.3) | 4 (23.5) | 6 (23.1) |  |
| PD-1/BV containing regimen | 17 (39.5) | 12 (70.6) | 5 (19.2) |  |
| Others | 16 (37.2) | 1 (5.9) | 15 (57.7) |  |
| **Response to 1st treatment (%)** |  |  |  |  |
| Satisfactory PR | 37 (17.9) | 25 (19.7) | 12 (15.0) | 0.503 |
| **Consolidation therapy** |  |  |  | **< 0.001** |
| Allo-HSCT | 15 (7.2) | 3 (2.4) | 12 (15.0) |  |
| Auto-HSCT | 58 (28.0) | 44 (34.6) | 14 (17.5) |  |
| w/o HSCT | 134 (64.7) | 80 (63.0) | 54 (67.5) |  |
| Chemo/RT/Novel agents | 36 (17.4) | 20 (15.7) | 16 (20.0) |  |
| Observation | 98 (47.3) | 60 (47.2) | 38 (47.5) |  |
| **Age at transplant, median (IQR), years** | 36 (28-46) | 40 (35-46) | 36 (24-46) | 0.310 |
| **Survival information** |  |  |  |  |
| Median follow-up (95%CI) | 46.1 (35.5-54.1) | 49.9 (36.7-60.9) | 37.6(29.6-56.3) | 0.340 |
| Median OS (95%CI) | 121 (98.7-NA) | 139 (98.7-NA) | 92.4(82.7-NA) |  |
| 3-year OS (95%CI) | 76.7% (70.2%-83.8%) | 79.9% (72.2%-88.4%) | 71.2% (60.1%-84.2%) |  |
| Median PFS (95%CI) | 82.7(44.7-NA) | 64.6 (41.2-NA) | 82.7 (39.2-NA) | 0.990 |
| 3-year PFS (95%CI) | 62.2% (55.3%-69.9%) | 62.8% (54.4%-72.4%) | 61.2% (50.0%-74.8%) |  |
| 3-year CIR | 33.6% | 37.0% | 27.2% | 0.155 |
| 3-year NRM | 4.7% | 0.8% | 7.3% | **0.004** |
| **Cause of death** |  |  |  | 0.062 |
| Primary disease | 45 (81.8%) | 30 (93.7%) | 15 (65.2%) |  |
| Infection | 5 (9.1%) | 0 (0.0%) | 5 (21.7%) |  |
| Other causes | 5 (9.1%) | 2 (6.3%) | 3 (13.1%) |  |

**CR:** Complete remission; **PR:** Partial remission; **SD:** Stable disease; **PD**: Progressive disease; **HSCT**: Hematopoietic stem cell transplantation; **Auto-HSCT**: Autologous hematopoietic stem cell transplantation; **Allo-HSCT**: Allogeneic hematopoietic stem cell transplantation; **IPI**: International Prognostic Index; **PIT**: Prognostic Index for T-cell lymphoma; **AITL**: Angioimmunoblastic T-cell lymphoma; **ALK-ALCL**: Anaplastic Lymphoma Kinase-negative Anaplastic Large Cell Lymphoma; **ALK+ALCL**: Anaplastic Lymphoma Kinase-positive Anaplastic Large Cell Lymphoma; **ANKL**: Aggressive NK-cell leukemia; **ENKTL**: Extranodal NK/T-cell lymphoma, nasal type; **HSTCL**: Hepatosplenic T-cell lymphoma; **PTCL-NOS**: Peripheral T-cell lymphoma, not otherwise specified; **CHOP**: Cyclophosphamide, doxorubicin, vincristine, prednisone; **PD-1/BV**: Programmed death-1/Brentuximab vedotin; **RT**: Radiation Therapy; **CNS**: Central nervous system; **UN**: Unknown; w/o: Without; **NA:** Not available; **OS**: Overall survival; **PFS**: Progression-free survival; **CIR**: Cumulative incidence of relapse; **NRM**: Non-relapse mortality; **CI**: Confidence Interval.

*"Others" entities included rare subtypes of PTCL, subcutaneous panniculitis-like T-cell lymphoma, enteropathy-associated T-cell lymphoma, and monomorphic epitheliotropic intestinal T-cell lymphoma, adult T-cell leukemia-lymphoma, primary cutaneous large anaplastic T-cell lymphoma, mycosis fungoides and anaplastic large cell lymphoma with unknown anaplastic lymphoma kinase status.

# Table S2. Patients' baseline characteristics for the first-line treatment non-responders

| **Characteristics** | **Overall**  n = 201 | **Nodal-PTCL**  n = 114 | **Non-nodal PTCL**  n = 87 | ***P* value** |
| --- | --- | --- | --- | --- |
| **Male (%)** | 127 (63.2) | 73 (64.0) | 54 (62.1) | 0.890 |
| **Age at diagnose, median (IQR), years** | 46 (34, 56) | 51 (36, 59) | 43 (29, 51) | **0.001** |
| **Disease (%)** |  |  |  | **< 0.001** |
| AITL | 38 (18.9) | 38 (33.3) | — |  |
| ALK-ALCL | 10 (5.0) | 10 (8.8) | — |  |
| ALK+ALCL | 7 (3.5) | 7 (6.1) | — |  |
| PTCL-NOS | 58 (28.9) | 58 (50.9) | — |  |
| ANKL | 8 (4.0) | — | 8 (9.2) |  |
| ENKTL | 33 (16.4) | — | 33 (37.9) |  |
| HSTCL | 26 (12.9) | — | 26 (29.9) |  |
| Other | 21 (10.4) | 1 (0.9) | 20 (23.0) |  |
| **Involvement of the spleen (%)** | 55 (61.1) | 25 (56.8) | 30 (65.2) | 0.548 |
| **Involvement of the bone marrow (%)** | 97 (48.3) | 58 (50.9) | 39 (44.8) | 0.479 |
| **Involvement of the CNS (%)** | 9 (4.5) | 3 (2.6) | 6 (6.9) | 0.269 |
| **ECOG score > 2 (%)** | 57 (28.4) | 34 (29.8) | 23 (26.4) | 0.771 |
| **B symptom (%)** | 133 (66.2) | 76 (66.7) | 57 (65.5) | 0.984 |
| **IPI score > 2 (%)** | 93 (46.3) | 47 (41.2) | 46 (52.9) | 0.134 |
| **PIT risk factors (%)** |  |  |  | 0.194 |
| 0-1 | 101 (50.2) | 51 (44.7) | 50 (57.5) |  |
| 2 | 64 (31.8) | 41 (36.0) | 23 (26.4) |  |
| 3-4 | 36 (17.9) | 22 (19.3) | 14 (16.1) |  |
| **Response to 1^st^ line treatment (%)** |  |  |  | **0.001** |
| Unsatisfactory PR | 40 (19.9) | 33 (28.9) | 7 (8.0) |  |
| SD | 45 (22.4) | 22 (19.3) | 23 (26.4) |  |
| PD or CR but early relapse | 116 (57.7) | 59 (51.8) | 57 (65.5) |  |
| **HSCT following 2^nd^ line treatment** |  |  |  | **< 0.001** |
| Auto-HSCT | 18 (9.0) | 14 (12.3) | 4 (4.6) |  |
| Allo-HSCT | 25 (12.4) | 6 (5.3) | 19 (21.8) |  |
| w/o HSCT | 158 (78.6) | 94 (82.5) | 64 (73.6) |  |
| **Disease status before HSCT (%)** |  |  |  | 0.956 |
| CR/PR | 34 (79.1) | 17 (85.0) | 17 (73.9) | 0.373 |
| SD/PD | 9 (20.9) | 3 (15.0) | 6 (26.1) |  |
| **Age at transplantation, median (IQR), years** | 41 (32, 49) | 42 (35, 47) | 41 (29, 52) | 0.800 |

**ECOG:** Eastern Cooperative Oncology Group**.**

Significant results (*P* value < 0.05) are bolded.

# Table S3. Patients' baseline characteristics for HSCT after the third-line and beyond treatment

| **Characteristics** | **Overall**  n = 31 | **Nodal-PTCL**  n = 19 | **Non-nodal PTCL**  n = 12 | ***P* value** |
| --- | --- | --- | --- | --- |
| **Male (%)** | 17 (54.8) | 10 (52.6) | 7 (58.3) | > 0.999 |
| **Age at diagnose, median (IQR), years** | 44 (31, 51) | 46 (38, 51) | 36 (27, 50) | 0.372 |
| **HSCT following 2^nd^ line treatment** |  |  |  | 0.140 |
| Allo-HSCT | 21 (67.7) | 11 (57.9) | 10 (83.3) |  |
| Auto-HSCT | 10 (32.3) | 8 (42.1) | 2 (16.7) |  |
| **Age at transplantation, median (IQR), years** | 44.0 (32.0, 49.0) | 45.0 (38.5, 48.0) | 38.0 (29.3, 52.3) | 0.778 |
| **Disease status before HSCT** |  |  |  | 0.337 |
| CR/PR | 18 (58.1) | 13 (68.4) | 5 (41.7) |  |
| SD/PD | 11 (35.5) | 5 (26.3) | 6 (50.0) |  |
| UN | 2 (6.5) | 1 (5.3) | 1 (8.3) |  |
| **Survival information** |  |  |  |  |
| Median follow-up (95%CI) | 53.1 (20.1-NA) | 27 (18.1-NA) | NA (54.7-NA) | 0.180 |
| Median OS (95%CI) | 20.4 (11.3-NA) | NA (17.9-NA) | 9.0 (4-NA) | **0.025** |
| 3-year OS (95%CI) | 48.2 (32.3-72.1) | 62.9% (43.1%-91.9%) | 28.1% (10.9%-72.9%) |  |
| Median PFS (95%CI) | 11.3 (6.7-NA) | 33 (12.7-NA) | 3.0 (1.8-NA) | **< 0.001** |
| 3-year PFS (95%CI) | 28.2 (14.2-55.8) | 38% (95% CI 17.9%-80.6%) | 10% (95% CI 1.6%-63.1%) |  |

Significant results (*P* value < 0.05) are marked in bold format.

# Table S4. Baseline for PTCL responders with nodal subtypes before and after propensity score matching

|  | **Before PSM** | | | |  | **After PSM** | | | |
| --- | --- | --- | --- | --- | --- | --- | --- | --- | --- |
| **Characteristics** | **Overall**  n = 124 | **Auto-HSCT**  n = 44 | **w/o HSCT**  n = 80 | ***P* value** |  | **Overall**  n = 60 | **Auto-HSCT**  n = 30 | **w/o HSCT**  n = 30 | ***P* value** |
| **Male, n (%)** | 81 (65.3) | 30 (68.2) | 51 (63.7) | 0.765 |  | 36 (60.0) | 20 (66.7) | 16 (53.3) | 0.429 |
| **Age at diagnose, median (IQR), years** | 46 (34, 60) | 45 (35, 55) | 52 (34, 62) | 0.094 |  | 45 (35, 56) | 44 (37, 57) | 47 (34, 56) | 0.510 |
| **Disease, n (%)** |  |  |  | 0.081 |  |  |  |  | 0.905 |
| AITL | 41 (33.1) | 21 (47.7) | 20 (25.0) |  |  | 21 (35.0) | 11 (36.7) | 10 (33.3) |  |
| ALK-ALCL | 10 (8.1) | 2 (4.5) | 8 (10.0) |  |  | 3 (5.0) | 2 (6.7) | 1 (3.3) |  |
| ALK+ALCL | 31 (25.0) | 7 (15.9) | 24 (30.0) |  |  | 14 (23.3) | 7 (23.3) | 7 (23.3) |  |
| PTCL-NOS | 41 (33.1) | 14 (31.8) | 27 (33.8) |  |  | 22 (36.7) | 10 (33.3) | 12 (40.0) |  |
| Others | 1 (0.8) | 0 (0.0) | 1 (1.2) |  |  | 0 (0.0) | 0 (0.0) | 0 (0.0) |  |
| **Year of diagnosis (2019-2022), n (%)** | 50 (40.3) | 20 (45.5) | 30 (37.5) | 0.501 |  | 22 (36.7) | 11 (36.7) | 11 (36.7) | > 0.999 |
| **PR to 1st line treatment, n (%)** | 24 (19.4) | 11 (25.0) | 13 (16.2) | 0.346 |  | 14 (23.3) | 8 (26.7) | 6 (20.0) | 0.760 |
| **IPI score > 2 (%)** | 38 (30.6) | 15 (34.1) | 23 (28.7) | 0.679 |  | 18 (30.0) | 11 (36.7) | 7 (23.3) | 0.398 |
| **PIT risk factors (%)** |  |  |  | 0.116 |  |  |  |  | 0.811 |
| 0-1 | 77 (62.1) | 26 (59.1) | 51 (63.7) |  |  | 37 (61.7) | 18 (60.0) | 19 (63.3) |  |
| 2 | 31 (25.0) | 15 (34.1) | 16 (20.0) |  |  | 16 (26.7) | 9 (30.0) | 7 (23.3) |  |
| 3-4 | 16 (12.9) | 3 (6.8) | 13 (16.2) |  |  | 7 (11.7) | 3 (10.0) | 4 (13.3) |  |
| **Involvement of the spleen (%)** |  |  |  | **0.002** |  |  |  |  | 0.551 |
| No | 94 (75.8) | 26 (59.1) | 68 (85.0) |  |  | 15 (25.0) | 21 (80.0) | 24 (80.0) |  |
| Yes | 29 (23.4) | 18 (40.9) | 11 (13.8) |  |  | 15 (25.0) | 9 (30.0) | 6 (20.0) |  |
| UN | 1 (0.8) | 0 (0.0) | 1 (1.2) |  |  | 0 (0.0) | 0 (0.0) | 0 (0.0) |  |
| **Involvement of the bone marrow (%)** | 50 (40.3) | 22 (50.0) | 28 (35.0) | 0.150 |  | 24 (40.0) | 10 (33.3) | 14 (46.7) | 0.429 |
| **Involvement of the CNS (%)** | 1 (0.8) | 0 (0.0) | 1 (1.2) | > 0.999 |  | 1 (1.7) | 0 (0.0) | 1 (3.3) | > 0.999 |

Propensity score matching (PSM) was performed to reduce potential confounding factors and ensure balanced covariates between the auto-HSCT and non-HSCT groups among nodal responders, with caliper of 0.05. The selected covariates were based on prior research and clinical expertise and included age at diagnosis, disease subtypes, year of diagnosis, bone marrow involvement, and PIT score.

Significant results (*P* value < 0.05) are marked in bold format.

**Table S5**. **Baseline for PTCL responders with non-nodal subtypes before and after propensity score matching**

| **Characteristics** | **Before PSM** | | | |  | **After PSM** | | | |
| --- | --- | --- | --- | --- | --- | --- | --- | --- | --- |
|  | **Overall**  n = 26 | **Allo-HSCT**  n = 12 | **Auto-HSCT**  n = 14 | ***P* value** |  | **Overall**  n = 16 | **Allo-HSCT**  n = 8 | **Auto-HSCT**  n = 8 | ***P* value** |
| **Male, n (%)** | 9 (34.6) | 2 (16.7) | 7 (50.0) | 0.171 |  | 4 (25.0) | 0 (0.0) | 4 (50.0) | 0.083 |
| **Age at diagnose, median (IQR), years** | 36 (24, 49) | 34 (23, 39) | 47 (33, 52) | 0.142 |  | 37 (30, 49) | 36 (30, 48) | 42 (30, 52) | 0.599 |
| **Disease, n (%)** |  |  |  | 0.333 |  |  |  |  | 0.842 |
| ANKL | 2 (7.7) | 2 (16.7) | 0 (0.0) |  |  | 0 (0.0) | 0 (0.0) | 0 (0.0) |  |
| ENKTL | 14 (53.8) | 5 (41.7) | 9 (64.3) |  |  | 7 (43.8) | 4 (50.0) | 3 (37.5) |  |
| HSTCL | 5 (19.2) | 2 (16.7) | 3 (21.4) |  |  | 5 (31.2) | 2 (25.0) | 3 (37.5) |  |
| Other | 5 (19.2) | 3 (25.0) | 2 (14.3) |  |  | 4 (25.0) | 2 (25.0) | 2 (25.0) |  |
| **Year of diagnosis (2019-2022), n (%)** | 14 (53.8) | 5 (41.7) | 9 (64.3) | 0.448 |  | 7 (43.8) | 4 (50.0) | 3 (37.5) | > 0.999 |
| **PR to 1st line treatment, n (%)** | 7 (26.9) | 3 (25.0) | 4 (28.6) | > 0.999 |  | 4 (25.0) | 2 (25.0) | 2 (25.0) | > 0.999 |
| **IPI score > 2 (%)** | 13 (50.0) | 7 (58.3) | 6 (42.9) | 0.694 |  | 8 (50.0) | 5 (62.5) | 3 (37.5) | 0.617 |
| **PIT risk factors (%)** |  |  |  | 0.634 |  |  |  |  | 0.809 |
| 0-1 | 17 (65.4) | 8 (66.7) | 9 (64.3) |  |  | 11 (68.8) | 6 (75.0) | 5 (62.5) |  |
| 2 | 6 (23.1) | 2 (16.7) | 4 (28.6) |  |  | 3 (18.8) | 1 (12.5) | 2 (25.0) |  |
| 3-4 | 3 (11.5) | 2 (16.7) | 1 (7.1) |  |  | 2 (12.5) | 1 (12.5) | 1 (12.5) |  |
| **Involvement of the spleen (%)** | 11 (42.3) | 6 (50.0) | 5 (35.7) | 0.736 |  | 6 (37.5) | 3 (37.5) | 3 (37.5) | > 0.999 |
| **Involvement of the bone marrow (%)** | 13 (50.0) | 8 (66.7) | 5 (35.7) | 0.238 |  | 7 (43.8) | 4 (50.0) | 3 (37.5) | > 0.999 |
| **Involvement of the CNS (%)** | 1 (3.8) | 1 (8.3) | 0 (0.0) | 0.937 |  | 16 (100.0) | 8 (100.0) | 8 (100.0) | > 0.999 |

Clinical characteristics were not significantly different between the allo-HSCT and auto-HSCT groups among non-nodal responders. PSM was also conducted to rigorously analyze the patients involved as a sensitivity analysis. Due to the limited sample size, a caliper of 0.2 was used.

# Table S6. Non-relapse mortality (NRM) rates of allo-HSCT in treating PTCL according to the literature review

| **First Author** | **Journal** | **Publication Year** | **Sample size** | **NRM** | **PMID** |
| --- | --- | --- | --- | --- | --- |
| **Corradini P** | J Clin Oncol | 2004 | 17 | 2y-NRM:6% | 15169805 |
| **Murashige N** | Br J Haematol | 2005 | 22 | 2y-NRM:28.5% | 16098071 |
| **Feyler S** | Bone Marrow Transplant | 2007 | 18 | 3y-NRM:39% | 17589529 |
| **Jacobsen ED** | Ann Oncol | 2011 | 52 | 3y-NRM: 27% | 21252059 |
| **Zain, J** | Leukemia & Lymphoma | 2011 | 37 | 5y-NRM:28.9% | 21699453 |
| **Dodero A** | Leukemia | 2012 | 52 | 5y-NRM:12% | 21904377 |
| **Zhou Y** | Zhongguo Shi Yan Xue Ye Xue Za Zhi | 2015 | 22 | 3y-TRM:48.7% | 26117014 |
| **Wang QL** | Zhonghua Xue Ye Xue Za Zhi | 2016 | 21 | 5y-TRM:41.8% | 27995879 |
| **Huang H** | Biol Blood Marrow Transplant | 2017 | 24 | 1y-NRM:18% | 28478121 |
| **Huang H** | Biol Blood Marrow Transplant | 2017 | 24 | 1y-NRM:18% | 28478121 |
| **Mussetti A** | Bone Marrow Transplant | 2019 | 45 | 2y-NRM:22% | 30531956 |
| **Modi D** | Leuk Res | 2019 | 39 | 5y-NRM:43.8% | 31228653 |
| **Z. Gu** | Annals of Hematology | 2019 | 21 | 3y-NRM:24% | 30539277 |
| **Epperla N** | J Hematol Oncol. | 2019 | 249 | 1y-NRM:19%; 2y-NRM:31%; 3y-NRM:33%,4y-NRM:36% | 30630534 |
| **De Latour R** | J Hematol Oncol. | 2020 | 285 | 1y-TRM:21%; 2y-TRM: 24%; 4y-TRM:28% | 32429979 |
| **Zhenyang G** | Cell Transplant | 2021 | 52 | 3y-NRM:21-22% | 33745341 |
| **Gu ZY** | Chin Med J (Engl) | 2021 | 56 | 3y-NRM:27% | 34133361 |
| **Schmitz N** | Blood | 2021 | 26 | 3y-NRM:31% | 33512419 |
| **Castagna L** | Bone Marrow Transplant | 2021 | 68 | 4y-NRM:9% | 33191403 |
| **Cornillon J** | Bone Marrow Transplant | 2021 | 41 | 21m-NRM:8% | 34363006 |
| **Novelli S,** | Transplant Cell Ther | 2021 | 201 | 1y-NRM:21.9% | 33857447 |
| **Hamadani M** | Blood Adv | 2022 | 1942 | 3y NRM:21-24% | 34861680 |
| **Savani M** | Br J Haematol. | 2022 | 803 | 1y-NRM:27%-28%; 3y-NRM: 30-31%; 5y-NRM:31% | 35106754 |
| **Berning P** | Leukemia | 2023 | 135 | 1y-NRM:14.8% | 37157017 |
| **Klimentova M** | Transplant Cell Ther | 2024 | 19 | 5y-NRM:5% | 38286354 |
| **Moser O** | Bone Marrow Transplant | 2024 | 46 | 5y-NRM:19.8% | 38331982 |

NRM: Non-Relapse Mortality

**References**

1. Swerdlow SH, Campo E, Pileri SA, Harris NL, Stein H, Siebert R, et al. The 2016 revision of the World Health Organization classification of lymphoid neoplasms. Blood. 2016;127(20):2375-90. Epub 20160315. doi: 10.1182/blood-2016-01-643569.

2. The International Non-Hodgkin's Lymphoma Prognostic Factors Project. A predictive model for aggressive non-Hodgkin's lymphoma. N Engl J Med. 1993;329(14):987-94. doi: 10.1056/nejm199309303291402.

3. Gallamini A, Stelitano C, Calvi R, Bellei M, Mattei D, Vitolo U, et al. Peripheral T-cell lymphoma unspecified (PTCL-U): a new prognostic model from a retrospective multicentric clinical study. Blood. 2004;103(7):2474-9. Epub 20031126. doi: 10.1182/blood-2003-09-3080.

4. Cheson BD, Fisher RI, Barrington SF, Cavalli F, Schwartz LH, Zucca E, Lister TA. Recommendations for initial evaluation, staging, and response assessment of Hodgkin and non-Hodgkin lymphoma: the Lugano classification. J Clin Oncol. 2014;32(27):3059-68. doi: 10.1200/jco.2013.54.8800.

5. Bellei M, Foss FM, Shustov AR, Horwitz SM, Marcheselli L, Kim WS, et al. The outcome of peripheral T-cell lymphoma patients failing first-line therapy: a report from the prospective, International T-Cell Project. Haematologica. 2018;103(7):1191-7.

6. Fine JP, Gray RJ. A Proportional Hazards Model for the Subdistribution of a Competing Risk. Journal of the American Statistical Association. 1999;94(446):496-509. doi: 10.1080/01621459.1999.10474144.

7. Sekhon JS. Multivariate and Propensity Score Matching Software with Automated Balance Optimization: The Matching package for R. Journal of Statistical Software 2011; 42 (7): 1 - 52. doi: 10.18637/jss.v042.i07.
